# The *Grande Rose* of the Reims Cathedral: an eight-century perspective on the colour management of medieval stained glass

**DOI:** 10.1038/s41598-019-39740-y

**Published:** 2019-03-01

**Authors:** Natan Capobianco, Myrtille O. J. Y. Hunault, Sylvie Balcon-Berry, Laurence Galoisy, Dany Sandron, Georges Calas

**Affiliations:** 1 Sorbonne Université, Muséum National d’Histoire Naturelle, UMR CNRS 7590, IRD, Institut de Minéralogie, de Physique des Matériaux et de Cosmochimie, IMPMC, Paris, 75005 France; 2 SOLEIL synchrotron, L’Orme des Merisiers Saint-Aubin BP48, 91192 Gif-sur-Yvette, France; 3 Sorbonne Université, UMR CNRS 8150, Centre André Chastel, Galerie Colbert, 75002 Paris, France

## Abstract

The *Grande Rose* of Reims Cathedral (France), a UNESCO Cultural Heritage Monument from the 13^th^ century, underwent several restoration works during the 20^th^ century. Its colours result from centuries of colour management from which little information remain. We used non-destructive and portable optical absorption spectroscopy to quantify glass colour and determine the colouring species on a large-scale study of this monumental window. We found six distinct colour groups, each containing both medieval and modern glasses, with colouring processes specific to each colour. This illustrates medieval glassmakers’ mastering of glass colouring and modern glassmakers’ management to reproduce medieval glasses colours. Full UV-visible-NIR energy range is necessary for determining the contribution of colouring elements as Fe^2+^ and Cu^2+^. Systematic thickness measurements reveal an average glass thickness of 3 mm and demonstrate the major control of chromophore concentration on glass colour. Yellow, red and purple colours arise from a single chromophore each, suggesting the use of well-defined glassmaking techniques leading to robust colour reproducibility. By contrast, blue and green glasses show different chromophore combinations depending on production time, which suggests more diversity in glassmaking techniques.

## Introduction

Stained glasses are one of the most impressive features of the medieval gothic architecture. From the 12^th^ century onwards^1,2^, their use has spread with the building of the greatest cathedrals of Western Europe, as a response to the increasing size of windows in these buildings, serving as iconographic supports and filling with light and colour the medieval churches. 250,000 m^2^ of glasses were manufactured in the 13^th^ century alone, among which only remain 15,000 m^2^ ^3^. Vivid and brilliant colours having a specific symbolism were obtained by intentionally adding chromophores during glassmaking. In the 19^th^ century, began a vast campaign of restoration and promotion of medieval monuments, unfortunately more aimed to the aesthetics of artworks than to the saving of the original materials. Ancient glasses were replaced by modern glasses, often having different compositions^1^. This compositional change modifies chromophore properties^2^ and the colour of modern and medieval glasses from Northern Europe may differ. Although glass colour is a major artistic property, only few quantitative measurements of the colour of stained glasses are available^2,4,5^, which limits our knowledge on the control and choice of these colours across centuries.

The diversity of colours in glasses arises from the absorption of visible light by transition metal ions intentionally added or inherited from raw materials^6^. The speciation (oxidation state and coordination number) of these ions vary with glass chemical composition and fabrication conditions, mostly furnace temperature and atmosphere, and control the resulting colour allowing the glassmaker to manage the final colour of the glass. X-ray absorption spectroscopy is most used to study the transition metal ions speciation^7–10^. The analysis of glass colour can help determine the nature of the chromophore, and may provide indirect indications on the chemical composition, dating and authenticity of glass pieces^11,12^ and the glassmaking techniques^4,8^. Optical absorption spectroscopy (OAS) allows a non-invasive identification of the chromophores responsible for glass colouring and portable methods have been developed to analyse the glasses without dismantling the panels^11,13^. Colorimetric coordinates may be derived from optical spectra, providing a quantitative assessment of the colour, which is otherwise a subjective criterion. As predicted by Beer-Lambert law, glass absorbance is also dependent on the thickness of the glass pieces, a parameter that needs to be independently measured by minimal-contact techniques on historical pieces. The absorbing properties of medieval stained glasses also determine light transmission inside Gothic monuments and influence human visual perception. Brighter colours during the 12^th^ century and more translucent glasses of the 15^th^ and 16^th^ centuries both admitted more light compared to 13^th^-century glasses^14^. A recent modelling suggests that the change to translucent glasses could be a response to climate change. Indeed, modifications in the North Atlantic Oscillations induced a transition from warm and sunny to cooler and cloudier conditions during the transition from the Medieval Warm Period to the Little Ice Age^15^.

**Figure 1:**
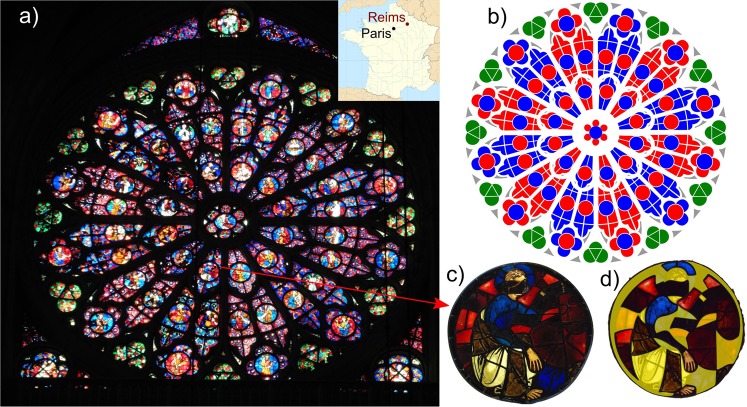
(**a**) The Grande Rose of the Reims Cathedral, France (inset). (**b**) Design pattern of the background colour of the panels of the Rose of Reims. (**c**) Figurative panel H1 showing blue, red, colourless and purple glasses. (**d**) Authenticity screening of panel H1. Yellow-shaded glasses are identified as modern, others dating from 13^th^ century.

The Gothic cathedral of Reims, a UNESCO Cultural Heritage Monument, is one of the most ambitious Gothic cathedral in France, being the coronation site of thirty French kings. Its stained glasses are a reference in the medieval art and have been extensively investigated^16,17^. However, they suffered major damage during World War I, with a major fire of the Northern tower in September 1914, after which only survived 1,500 m^2^ of stained glasses from the original 3,900 m^2^ still remaining at the beginning of the 20^th^ century. Facing to West, the Grande Rose closes the largest bay of the cathedral (Fig. 1a). It has been originally built around 1280 AD and is constituted of tens of thousands of glass pieces assembled in 492 panels occupying 80 windows. More detailed history of the Rose is given in Supplementary Information. The Rose follows an elaborated pattern (Fig. 1b) with a background colour pattern which consists in a double scansion of red and blue background panels, from centre to outside and going around the centre. As for other Middle Age roses, the glass areas of the rose of Reims follow a fractal pattern, which was interpreted as indicating a design with the same roughness model for solid (i.e. masonry) areas and glass areas^18^. One peculiar feature of the Grande Rose of Reims cathedral is the use of the green colour in background of trefoils that form the outermost ring of the pattern, while backgrounds were commonly blue and red at this time^19,20^. This peculiar use of green colour on the outermost ring may be motivated by forming a transition between the stained glasses and the outer stone ring, decorated with vegetal sculptures which were probably originally painted.

Several restorations of the cathedral of Reims occurred from the 16^th^ century to present, with a replacement of some ancient glasses by modern ones. In addition, most stained glasses of the lower level have been suppressed in the 18^th^ century in order to increase the lighting of the cathedral. The surviving glasses illustrate the typical variety of colours obtained during the 13^th^ century and are a major witness of Gothic medieval stained glasses that underwent various steps of colour management throughout eight centuries. The contribution of the various colours used for restoration is similar to those used in the 13^th^ century. Distinguishing ancient from modern glasses among the investigated pieces was made by a critical visual analysis of the glasses and overlying paintings (grisaille) and comparison with other historical glasses (Fig. 1c,d). A new restoration campaign has been recently completed in 2016 and we performed a colorimetric analysis of the Grande Rose, 12 m diameter, by analysing 101 pieces of glass from 20 panels, chosen for their diversity of colour and estimated age (the repartition of glasses analysed by colour and age is given on Supplementary Fig. S2, detailed count is given in Supplementary Table S1). Investigating the various chromophores used for glassmaking provides us with a unique overview of this major artwork and the techniques of glass colour production and use from Middle Age to modern era. This provides information on how medieval craftsmen managed the colour of glasses during the creation of the Rose and how modern glassmakers adapted their techniques to better mimic medieval glasses during the restoration of the Rose.

## Results

### Glass thickness

**Figure 2:**
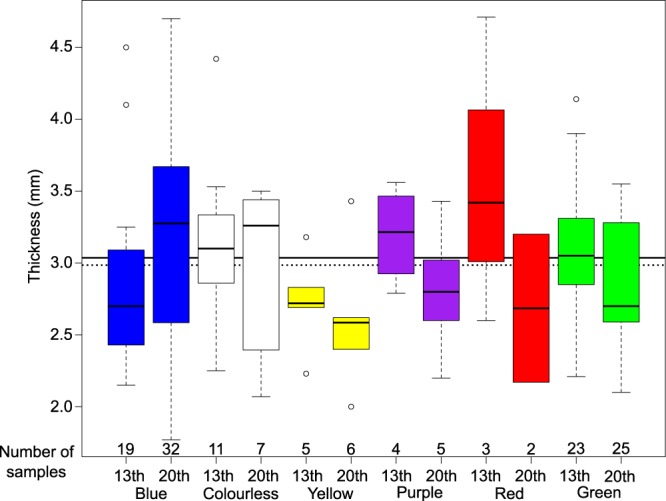
Box-and-whisker plot of the thickness of the stained glasses from Reims Cathedral, according to age and colour compared to the overall thickness average of medieval (solid line in background) and modern glasses (dashed line). The box is limited by the first and third quartiles of the data distribution. The “whiskers” represent the last value before 1.5 times midrange (the arithmetic mean of maximum and minimum values) beyond the first or third quartile. Circles represent data lying outside of the whiskers.

Glass thickness helps ensure window sturdiness, minimising the window weight and in addition optimising light transmission. As light absorption is proportional to chromophore concentration and glass thickness, we have measured the glass thickness prior to each spectroscopy measurement, at the spot analysed by optical spectroscopy, using a minimal-contact ultrasonic gauge. The overall average thickness of the medieval and modern glasses is similar: 3.03 mm and 2.96 mm respectively (Fig. 2). These values are in agreement with the few data on flat glasses of similar age, in the range 1.5–3 mm, such as in France^4,13^, Germany^21^, Italy^22^ or Spain^23,24^.

Close thickness values are observed for the different colours and suggest the use of high quality, thin glass in agreement with the royal status of this cathedral. The small deviation between the thickness of medieval and modern glasses complies with the restoration process of replacing sparse glass pieces by bridging ancient and new pieces with the lead bonds. Most glass pieces have non-uniform thickness over their surface. However this thickness variation cannot be assigned to the glass flowing since it has been demonstrated that the maximum flow is estimated to ca. 1 nm over a billion year^25,26^. The shape of thickness inhomogeneities suggests that most glasses have been blown as cylinders to obtain broad sheet glasses, but some medieval glasses are crown glasses.

Yellow, purple, red and green modern glasses show a similar thickness and are thinner than the ancient ones. Medieval and modern colourless glasses have similar thicknesses. Medieval red glasses are the thickest ancient ones (3.58 mm on average), because they were produced as flashed (i.e. double-layered) glasses. On the contrary, in the case of blue glasses, most medieval ones are thinner than 3 mm (2.88 mm on average) and than the blue modern glasses. However, as we measured only the thickness of the glasses analysed with OAS, the thickest blue glasses having an absorbance higher than 3 are not considered here. This suggests that the medieval blue glasses were more concentrated than the modern ones.

### Colorimetric characterisation

**Figure 3:**
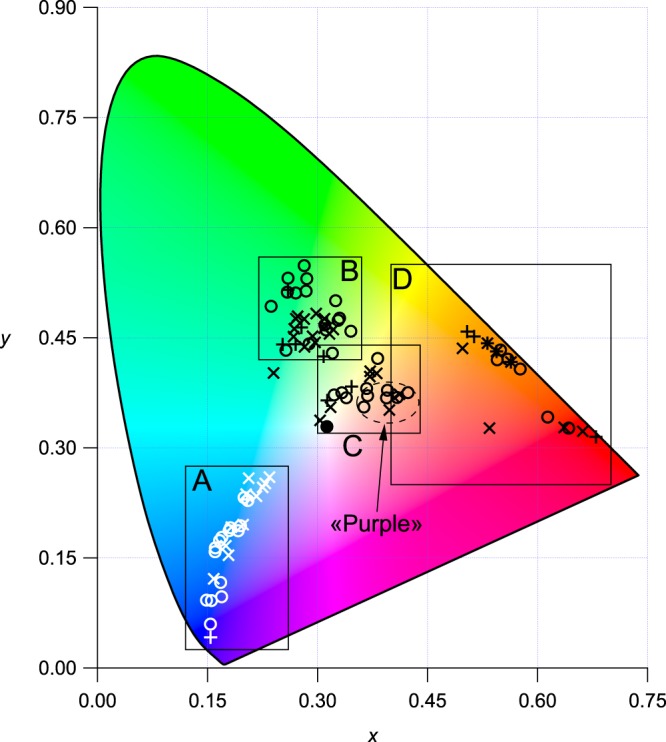
CIE*xy* chromaticity diagram of the colour of all the studied samples. ○: modern glasses, ×: medieval glasses. Blue glasses have a white marker for legibility reasons. The black line delimits the area of the plot which is accessible by any of the colours the human eye can see: monochromatic loci and the “purple” line. The black point in the centre have the coordinates of the white point of the diagram. Regions in rectangles will be zoomed in the following figures. Ellipse in region C represents colour of so-called *purple* glasses.

In Fig. 3, the colorimetric *x* and *y* coordinates of the investigated glasses are overlaid onto the CIE (Commission Internationale de l’Eclairage) 1931 colour space template, on which any point in the *xy* diagram gives the chromaticity (hue and saturation) of the colour^27^. In this representation, the monochromatic (or fully saturated) colours lie on the horse-shoe shaped locus line defining all visible light wavelengths. At the centre of this diagram, the white reference point enables the assessment of the colour saturation through the distance of the experimental colour to the white point. The colours of the stained glasses we have investigated are not intermixed and span over six distinct regions.

#### Blue glasses

**Figure 4:**
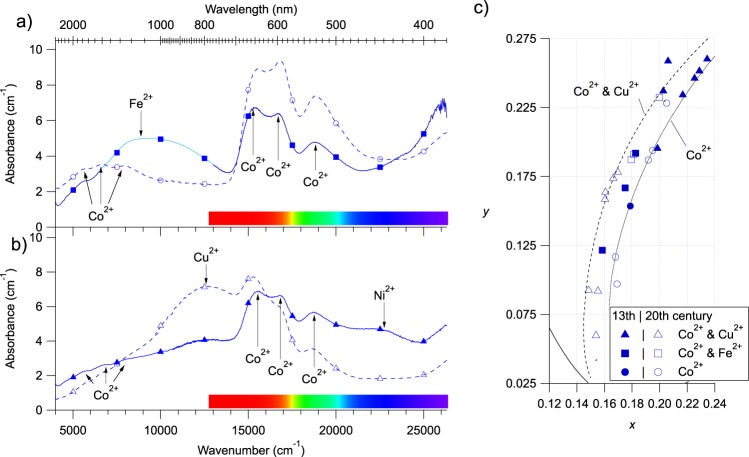
(**a**,**b**) Thickness normalised optical absorption spectra of the three main types of blue modern (dashed lines) and medieval (solid lines) glasses. Unless explicitly mentioned, spectra are not corrected nor smoothed. Markers refer to the nature of the chromophores (see inset legend of c and main text). (**a**) Co^2+^-alone and Co^2+^-Fe^2+^ glasses. The lighter coloured line has been smoothed, the original spectrum is given in Supplementary Fig. S11. (**b**) Examples spectra of Co^2+^-Cu^2+^ medieval and modern glasses. (**c**) Zoom on the CIE*xy* chromaticity diagram for blue glasses (region A of Figure 3). Lines are Beer-Lambert trend lines calculated for spectra representative of a chromophore compositions. Their computation is explained in the methods.

The optical absorption spectra of the 28 studied blue glasses show the characteristic absorption bands of Co^2+^ ^8^ (two triplets at 534, 599, 647 nm and 1256, 1510, 1764 nm), cobalt being reported as the main blue chromophore in medieval glasses^4,8,12,28^. These spectra can be grouped into three types, determined by the position of characteristic absorption bands (Fig. 4a,b): (i) Co^2+^ is the only chromophore; (ii) Co^2+^ coexists with Fe^2+^ (about 25% of analysed glasses), from iron impurities in the raw materials or intentionally added; (iii) Co^2+^ coexists with Cu^2+^ (about 43% of analysed glasses) arising from a voluntary addition. These three types of spectra are found in both medieval and modern glasses. In addition, all medieval Co^2+^-Cu^2+^ glasses show a broad, featureless absorption band at 440 nm assigned to Ni^2+^, also present in Co^2+^-alone modern glasses. The Co^2+^-alone blue glasses are blue-purple, while the presence of Cu^2+^ or Fe^2+^ gives a more greenish hue: this is consistent with the modification of the transmission window in the red range by the absorption bands of Cu^2+^ and Fe^2+^ centred at 780 nm and 1050 nm, respectively (Fig. 4c).

The colour coordinates of the blue glasses (Fig. 4c) follow a regular trend from the white point towards the monochromatic locus line. We used the Beer-Lambert law to model the influence of variations of chromophore concentration or glass thickness on glass absorbance for Co^2+^-alone and Co^2+^-Cu^2+^ (with the highest Cu^2+^ contribution) blue glasses (Fig. 4c). These trend lines (their computation is explained in the methods) match most of the colour diversity and span two orders of magnitude of absorption variation. Co^2+^-Fe^2+^ samples lay between the two trends because of the presence of Fe^2+^, which absorbs red light less efficiently that Cu^2+^. Co^2+^-Cu^2+^ medieval glasses are close to the Co^2+^-alone trend, because of the weak absorption of red light due to a low Cu^2+^ concentration. Moreover these glasses are less saturated (i.e. closer to the white point) because of the presence of Ni^2+^ absorption band in the blue transmission window. As glass thickness only varies by ±20%, it is mainly the Co^2+^ concentration in the glass that drives the intensity variations of the blue colour of the Reims stained glasses.

Using the measured glass thickness and the molar extinction coefficients, the Beer-Lambert law provides with an estimate of the concentration of the colouring species (cf. Supplementary Information). When Co^2+^ is the only chromophore, its concentration is around 1600 ± 500 ppm. When Co^2+^ is not alone, the Co^2+^ content lies around 800 ± 400 ppm and thus is lower than in Co^2+^-alone blue glasses. The estimated Co^2+^ concentrations agree with results from the contemporary glasses of the Sainte-Chapelle in Paris (France). Modern Co^2+^-Cu^2+^ blue glasses contain at most 0.1 wt% Cu^2+^, which is too low to be assessed in medieval Co^2+^-Cu^2+^ glasses. The Fe^2+^ content is around 0.20 ± 0.04 wt% as in colourless glasses (see below), which indicates that the presence of Fe^2+^ is unintentional. The Co^2+^ visible absorption bands shift slightly (3 nm) to lower energies and gain intensity with the presence of Cu^2+^ and/or Fe^2+^ absorption bands (Supplementary Fig. S9), as a result of the overlap of the absorption band of Co^2+^ and the other colouring species. The use of the position of the Co^2+^ 534 nm band for deducing the chemical composition and dating of blue glasses^12^ should be considered with caution. We succeeded by using optical spectra extending to the near-infrared to detect the presence of ancillary colouring species that may affect the actual position of Co^2+^ absorption band. In the case of modern glasses, the three different combinations of blue chromophores are consistent with the fact that 19^th^-century glassmakers knew these three colouring recipes^29^: they were able to make iron-free glass, but sometimes also added iron on purpose to remove the red component of the blue colour obtained using cobalt alone. In the case of the Co^2+^-alone type, it is unlikely that medieval glassmakers were able to obtain a Co^2+^-alone glass, as traces of Fe^2+^ are expected in medieval glasses, and reveal a possible incorrect dating of the glass based on visual inspection. Co and Cu have been observed in the composition of 13^th^-century glasses from cathedrals of northern France^30^. Contrary to iron, copper is not naturally present in raw materials and was added on purpose. According to the OAS spectra, Cu^2+^ concentration was lower in medieval glasses than in modern ones. The presence of nickel in medieval Co^2+^-Cu^2+^ glasses suggests its introduction by cobalt raw materials^28,30^. However, Co^2+^ alone and Co^2+^-Fe^2+^ medieval glasses do not show the presence of nickel, which suggests that different cobalt sources have been used for making these medieval glasses.

#### Green glasses

**Figure 5:**
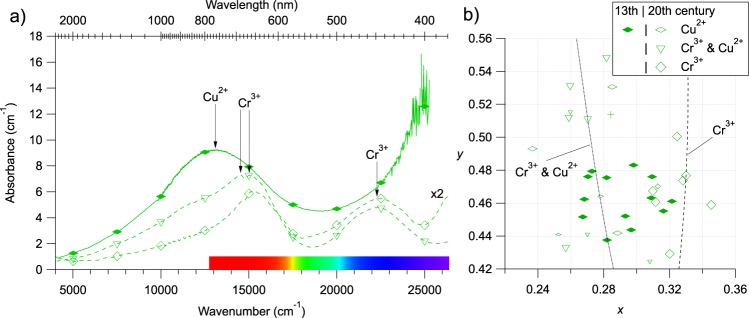
(**a**) Thickness normalised optical absorption spectra of the three main types of green modern (dashed lines) and medieval (solid lines) glasses. The spectrum with hollow square markers has been multiplied by a factor 2 for legibility reasons. Spectra are not corrected nor smoothed. (**b**) Zoom on the CIE*xy* plot of the green glasses (region B of Figure 3). Full markers are for medieval samples, hollow markers for modern samples. The Beer-Lambert-trend lines are computed as explained in the methods.

The 34 green glasses investigated also show contrasted OAS spectra (Fig. 5a): (i) half of the analysed glasses shows a single broad band at 780 nm assigned to Cu^2+^, at the same position as in the blue Co^2+^-Cu^2+^ glasses, and a strong and broad absorption band beginning at 500 nm, with maximum probably in the UV range, which origin may be assigned to a charge transfer between oxygen and Fe^3+^; (ii) 24% show the characteristic Cr^3+^ spectrum^6,13,31^ (a band at 475 nm and a doublet at 658 and 686 nm); (iii) 26% show absorption bands of Cr^3+^ and Cu^2+^. The different position of the transmission window in these three kinds of glasses gives a significant scatter of colorimetric coordinates (Fig. 5b). Cu^2+^-alone glasses have a more bluish hue relative to the Cr^3+^-alone glasses, with a transmission window at 520 and 560 nm, respectively, as Cu^2+^ and Cr^3+^ absorb in the red and the blue part of the spectrum, respectively. Cr^3+^-Cu^2+^ and Cu^2+^-alone glasses have similar hues, but the former have a higher saturation level because of a narrower transmission window. With the same formalism as for blue glasses, Beer-Lambert trend lines have been computed for Cr^3+^- alone and Cr^3+^-Cu^2+^ glasses, defining a domain in which most investigated glasses are located.

Using the same method as for the blue glasses, the concentration of Cu^2+^ and Cr^3+^ can be estimated from the absorption band intensity to ca. 0.6 ± 0.2 and 0.4 ± 0.1 wt% respectively (see Supporting Information Table S4). The concentration range for Cr^3+^ remains the same when both chromophores are used simultaneously, while Cu^2+^ concentration stays below 0.1 wt%. The concentration of Cu^2+^ (when alone) is at least 3 times lower than the typical concentration of total copper in medieval green glasses^32^. This is consistent with a Cu^2+^/Cu_total_ ratio around 0.3 in glasses melted in air^33^.

All chromium–coloured glasses of the Grande Rose of Reims are modern, which agrees with the fact that chromium pigments were not used in Europe before the 18^th^ century^34^. A few green glass samples were identified as modern with some uncertainty, but OAS analysis revealed that they were coloured only by Cu^2+^, suggesting a medieval origin.

#### Colourless and purple glasses

**Figure 6:**
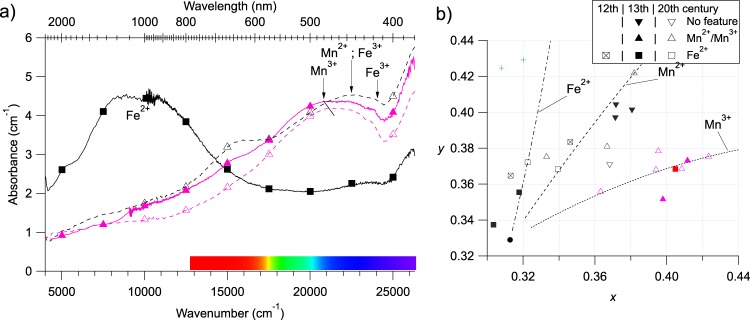
(**a**) Thickness normalised optical absorption spectra of colourless (black lines) and purple (purple lines), modern (dashed line) and medieval (solid line) glasses. Spectra are not corrected nor smoothed. Zoom on the 19000–26000 cm^−1^ range in given in Supplementary Fig. S10. (**b**) Zoom on the CIE*xy* chromaticity diagram region C of Figure 3. Full markers are for medieval samples, hollow markers for modern samples. The Beer-Lambert-trend lines are computed as explained in the methods.

The 13 colourless glasses show greenish to yellowish hues. The former are related to weak absorption bands from Fe^2+^ in the near IR^13^ (Fig. 6a) and from Fe^3+^ in the near-UV (423, 450 nm)^35^. The latter are mostly observed in modern glasses, showing a superposition of absorption bands in the blue-violet range: narrow bands from Mn^2+^ (450 nm) and Fe^3+^ with a broad band from Mn^3+^ ^6,36^ (around 480 nm). Residual colours come from residual impurities, either from the raw materials (mainly iron) or during glassmaking (presence of strongly colouring species like Co^2+^, contamination from melting pots…).

The 9 purple glasses show the broad asymmetric absorption band characteristic of Mn^3+^ characteristic of melting under oxidizing conditions^6^ (Fig. 6b). The maximum absorption shifts from 485 nm down to 471 nm from the medieval to the modern purple glasses, indicating a modification in the type of alkali used in the glass composition^37^. This observation is consistent with the medieval glasses having a potash-type composition, as the modern glasses have a soda-lime chemical composition^1^. All purple glasses also show features assigned to Mn^2+^ and Fe^3+^ ^35,36^.

The colour of both colourless and purple glasses relies on the control of the subtle redox equilibrium between iron and manganese during glassmaking. Ancient glassmakers used to add manganese to the glass to remove the greenish colour from Fe^2+^ impurities^7^, using the reaction Mn^3+^ + Fe^2+^ = Mn^2+^ + Fe^3+^. The shift of this redox equilibrium to the right transforms Mn^3+^ and Fe^2+^ into Mn^2+^ and Fe^3+^ (weak yellow-orange chromophores)^4,38,39^. When an excess of oxidizing manganese is added as all Fe^2+^ is oxidized into Fe^3+^, the remaining Mn^3+^ can intensely purple colour the glasses.

The colorimetric analysis (Fig. 6c) shows that purple glasses have a weak orange hue, which suits for displaying skin and flesh. They follow the Beer-Lambert trend line of Mn^3+^-coloured glasses. As the thickness of purple glasses only varies by about 20% (Fig. 2), the colour variation is due to variations of the manganese concentration. In modern colourless glasses and some medieval purple glasses, we observe an additional feature at 650 nm, which may be caused by Co^2+^ or Cr^3+^ ions (Fig. 6a,b). This agrees with the addition of blue and green glass cullets during the fabrication of colourless glasses for restoration purposes, as reported in the 19^th^ century^29^. In medieval times, these features may reveal the presence of impurities due to a reuse of the glassmaking pots.

The concentration of Fe^2+^ in colourless glasses is estimated around 0.2 ± 0.1 wt%, as in the blue glasses, which is lower than the typical concentration of total iron in colourless French medieval glasses (around 0.5 wt%)^8^. This indicates a majority of weakly colouring Fe^3+^ in these glasses^8,40^. By approximating the value of the absorption coefficient of Mn^3+^ for soda-lime-silica glasses^41^, the concentration of Mn^3+^ may be assessed to be ca. 0.2 ± 0.05 wt%. These values are lower than the typical manganese content in medieval purple glasses (around 1.5 wt%)^42^: here too, manganese occurs mostly as weakly colouring Mn^2+^ ^43^. Darkening induced by UV-light irradiation, also called “solarisation” and due to the photo-oxidation of Mn^2+^ to Mn^3+^, can be evidenced by comparing UV-exposed and non-exposed glass pieces, however this was not possible in the case of the present glasses. According to Long^44^, solarisation was observed in antimony containing glasses. However, European medieval glasses did not contain antimony^39^. Altogether, we could assume that solarisation and its effect on the glass color are neglectible in the present case.

#### Red and yellow glasses

**Figure 7:**
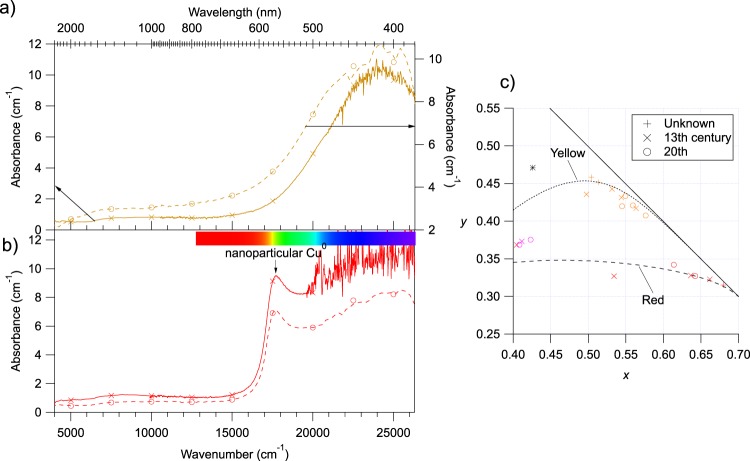
(**a**,**b**) Thickness normalised optical absorption spectra of yellow (**a**) and red (**b**), modern (dashed line) and medieval (solid line) glasses. Spectra are not corrected nor smoothed. Spectra become noisy towards high-energy range because of the high optical density of the samples, near the spectrometer detection limit. (**c**) CIE*xy* plot of the yellow and red glasses (Region D of Figure 3). ○: modern glasses, ×: medieval glasses, +: glasses of unknown age. * is an example colour of yellow silver stained glass from Molina *et al*.^5^. The Beer-Lambert-trend lines are computed as explained in the methods.

The 10 yellow glasses are bulk coloured and show similar OAS spectra characterized by a broad absorption band beginning at 600 nm with a maximum near 410 nm, devoid of the characteristic absorption features from Fe^3+^ (Fig. 7a). A broad, minor weak feature near 1000 nm indicates the presence of minor amounts of Fe^2+^. This excludes a yellow silver stain, characterised by a narrower absorption band peaking at near 470 nm^6,45^. This is consistent with the fact that this technique appeared in the 14^th^ century^46^, which is more recent than the fabrication of the Reims stained glasses. The similar spectral shape of ancient and modern glasses suggests the use of similar recipes and indicates that yellow sliver stained glasses were not used during modern restorations. The OAS spectra are consistent with the colouring species being the ferric iron-sulphide chromophore, characteristic of glassmaking under reducing conditions as encountered in the elaboration of amber glasses^47^.

The presence of chips at the surface of the investigated red glasses indicates that they were flashed onto a colourless glass and that they do not correspond to the multi-layered red glasses that have been recently described in glasses from the high medieval period^48^. The OAS spectra of red glasses (Fig. 7b) show the 566 nm band of the surface plasmon resonance from metallic Cu nanoparticles formed during glassmaking under reducing conditions^2,4,6^. In addition to this feature, some medieval glasses show the weak Fe^2+^ contribution at 1050 nm, that may be present in the colourless base glass prepared in normal melting conditions^6,8^.

Figure 7c shows that, whatever their age, both yellow and red glasses show highly saturated colours and follow their respective Beer-Lambert trend lines, which confirms that colour variation is not due to a change in the nature of chromophores. Both yellow and red glasses were complex to obtain and required a multi-step glass preparation. Nevertheless, the obtained colours are quite reproducible as shown by the small distribution of the colorimetric coordinates on the diagram. This suggests that variations in the colour recipe had little effect on the resulting colour or alternatively that these were very constrained recipes allowing little variation. Glassmaking treatise and textbooks suggest that red, yellow and purple colours required a high technical mastering of glassmaking, including advanced control of the chromophore (copper, iron, manganese) redox via the temperature of the glass and the kinetics of the process^4,48^. Hence our results suggest that the recipe for these colours was fixed.

## Discussion

The Rose of the Reims cathedral illustrates the glass colours used in the 13^th^ century. Even if medieval colours represented peculiar symbols, e.g. red for Christ’s colours or blue for the Virgin’s^49,50^, it remains unclear whether this symbolic extended to stained glass or if the choice of colours was rather dictated by fashion^51,52^. Thus blue, red and green were the only colours used for background. They have also been used for other purpose, for example for the characters’ clothes, together with yellow and colourless glasses. The only colour to have a specific use is flesh-purple, which was used exclusively for depicting the characters’ skins. Although different hues and saturation exist for each colour (especially for green and blue), our results show the absence of correlation between the choice and the use of the hue or saturation of a given colour. For instance, in the case of the blue colour, for which different saturations are easy to spot, the analysed glasses span both backgrounds and clothes, and show no specific use of the different saturations. On several panels, blue glasses of different saturations are even juxtaposed in a cloth or in background.

The resulting colour depends mainly on the concentration of the chromophore and the thickness of the glass. As we measured the thickness of the glasses, we are able to recalculate the chromatic coordinates for normalizing glasses thickness at 3 mm, which does not change the spread of the colour hues and saturation (Supplementary Fig. S8). This indicates that chromophore concentration controls the diversity of resulting colours. This was achieved via the glass composition (the recipe) and not during the blowing of the glass panes, which defines the final glass thickness.

In monuments like the Reims Cathedral, which have been frequently restored during centuries, we must wonder how well the restoration glasses fit in the original artwork. OAS evidenced in particular that colouring methods evolved for blue and green glasses. Colorimetry allows us to make a rigorous comparison between the colour of medieval and 20^th^-century glasses. For blue glasses, Co^2+^-alone restored glasses have a more purple hue and Co^2+^-Cu^2+^ restored glasses are slightly greener than the original glasses. The first difference is visual but the second is more difficult to spot, as the resulting spectrum is not so different between medieval and modern glasses. For green glasses, we observe a slight hue difference between modern Cr^3+^-alone glasses and ancient Cu^2+^-alone glasses, which have a bluer hue. However, this difference has been compensated by the addition of copper to the chromium coloured glass. Altogether, this demonstrates that modern glassmakers worried about making glasses for restoration with the same hue as the original medieval glasses. The addition of copper to the chromium-based glasses shifts the colour to a bluer hue, similar to the colour of an ancient glass thicker than usual. There is still a small difference in saturation between the colour of the medieval and modern glasses, making it possible to distinguish some modern glasses based on the sole colour criterion. We were also able to demonstrate that for other colours, no significant colour difference was observable between ancient and modern glasses. This is remarkable especially for colourless glasses, as medieval glassmakers were unable to produce proper colourless glasses, but modern glassmakers could, which implies that modern glassmakers had to care to make glasses retaining a medieval visually aspect. For example, there is evidence that aging treatments were used at the end of the 19^th^ century to create modern panels with a 13^th^ century look during the restoration of stained glass windows from the Troyes Cathedral^53,54^. 19^th^-century glassmakers^29^ intentionally degraded the quality of their colourless glasses to fit the original artwork, in order to reproduce the imperfection of medieval colourless glasses and not to make too obvious their interventions during the restoration of medieval stained glass windows.

## Methods

Critical observation determined the age of the glasses upon visual analysis of the glass and the paintings and comparison with other well-known glasses. The analysed glass pieces were chosen based on their colour and estimated date of fabrication in order to collect the most exhaustive corpus. Glass thickness was measured with a Olympus ultrasound gauge 45MG-X-MT-E by averaging 5 measures on the glass pieces investigated by optical spectroscopy, all 5 measures being carried at the spot analysed by optical absorption spectroscopy.

OAS spectra were acquired in transmission with a specific mobile set-up described elsewhere^13^. Here this set-up has been improved by adding a deuterium lamp STE-SL3 from Laser2000 as a light source for the near-UV range. The optical fibres used to carry the light from the sources to the collimating lens and from the collecting lens to the detectors are both Y-fibres made as double core in the single fibre and single core in each double fibre, which enables to transmit light simultaneously from both sources or to both spectrometers. All four fibres are multimodal and have 200 *μ*m core. Each Y-fibre is composed of one high-OH silica fibre protected against solarisation, for transmission in the 350–1000 nm energy range, and one low-OH silica fibre for transmission in the 400–2500 nm energy range. OAS spectra were acquired between 350 nm and 2500 nm (Supplementary Fig. S5). The importance of the near-UV range for colorimetry is discussed in detail in Supplementary Fig. S6.

Colorimetric *Yxy* coordinates were computed from the measured spectra using the CIE 1931 convention (see Supplementary Fig. S7) for further details). The calculations were made by interpolating the CIE 1931 observer standards $\bar{x}$, $\bar{y}$ and $\bar{z}$ and the D65 illuminant (average midday light in Western Europe) published by the CIE. Interpolation is needed because the observer standards have a 5 nm step size whereas the UV-visible spectrometer has 0.45 nm step size. By multiplying an optical spectrum with various factors from 0.04 to 25 and then calculating the *xy* coordinates, we are able to derive the trajectory of colour of samples with same chromophore concentrations and varying thickness or vice-versa. Such trajectories are called *Beer-Lambert trend lines*.

## Supplementary information

Supplementary Information

## References

[1] Rehren T, Freestone IC (2015). Ancient glass: From kaleidoscope to crystal ball. Journal of Archaeological Science.

[2] Meulebroeck, W., Wouters, H., Nys, K. & Thienpont, H. Authenticity screening of stained glass windows using optical spectroscopy. *Scientific Reports* **6**, 37726, 10.1038/srep37726 (2016). 10.1038/srep37726 PMC5121600 27883056

[3] Luczpinski, E. Le Vitrail par Eve Luszpinski, http://www.vitrail.free.fr/CSNV/dossiers/evelusz.php.

[4] Hunault, M. O. J. Y. *et al*. Nondestructive Redox Quantification Reveals Glassmaking of Rare French Gothic Stained Glasses. *Analytical Chemistry* **89**, 6278–649, 10.1021/acs.analchem.7b01452 (2017). 10.1021/acs.analchem.7b01452 PMC5645756 28494150

[5] Molina, G. *et al*. Color and dichroism of silver-stained glasses. *Journal of Nanoparticle Research* **15**, UNSP 1932, 10.1007/s11051-013-1932-7 (2013).

[6] Bamford, C. R. *Colour Generation and Control in Glass* (Elsevier Amsterdam, 1977).

[7] Quartieri, S., Riccardi, M. P., Messiga, B. & Boscherini, F. The ancient glass production of the Medieval Val Gargassa glasshouse: Fe and Mn XANES study. *Journal of Non-Crystalline Solids* **351**, 3013–3022, 10.1016/j.jnoncrysol.2005.06.046 (2005).

[8] Hunault, M. *et al*. Spectroscopic Investigation of the Coloration and Fabrication Conditions of Medieval Blue Glasses. *Journal of the American Ceramic Society* **99**, 89–97, 10.1111/jace.13783 (2016).

[9] Farges F, Etcheverry M-P, Scheidegger A, Grolimund D (2006). Speciation and weathering of copper in “copper red ruby” medieval flashed glasses from the Tours cathedral (XIII century). Applied Geochemistry.

[10] Ferrand, J. *et al*. Browning Phenomenon of Medieval Stained Glass Windows. *Analytical Chemistry* **87**, 3662–3669, 10.1021/ac504193z (2015). 10.1021/ac504193z 25688643

[11] Meulebroeck W (2011). Optical spectroscopy as a rapid and low-cost tool for the first-line analysis of glass artefacts: A step-by-step plan for Roman green glass. Journal of Archaeological Science.

[12] Ceglia A (2012). Using optical spectroscopy to characterize the material of a 16^th^ c. stained glass window. Integrated Approaches to the Study of Historical Glass - Ias12.

[13] Hunault, M. *et al*. Assessment of Transition Element Speciation in Glasses Using a Portable Transmission Ultraviolet-Visible-Near-Infrared (UV-Vis-NIR) Spectrometer. *Applied Spectroscopy* **70**, 778–784, 10.1177/0003702816638236 (2016). 10.1177/0003702816638236 26988660

[14] Simmons CT, Mysak LA (2010). Transmissive properties of Medieval and Renaissance stained glass in European churches. Architectural Science Review.

[15] Simmons CT, Mysak LA (2012). Stained Glass and Climate Change: How are they Connected?. Atmosphere-Ocean.

[16] Lillich, M. P. *The Gothic Stained Glass of Reims Cathedral* (Penn State Press, 2011).

[17] Balcon-Berry, S. Stained Glass and the Chronology of Reims Cathedral. In Nolan, K. & Sandron, D. (eds) *Arts of the Medieval Cathedrals*. *Studies on Architecture*, *Stained Glass and Sculpture in Honor of Anne Prache*, AVISTA Studies in the History of Medieval Technology, Science and Art, 10.4324/9781315262055-17 (2015).

[18] Samper A, Herrera B (2016). A Study of the Roughness of Gothic Rose Windows. Nexus Network Journal.

[19] Lafond, J. & Perrot, F. *Le Vitrail: origines*, *technique*, *destinées* (La Manufacture, 1988).

[20] Grodecki L, Brisac C (1984). Le Vitrail Gothique Au 13e Siècle.

[21] Hormes J (2013). Medieval glass from the Cathedral in Paderborn: A comparative study using X-ray absorption spectroscopy, X-ray fluorescence, and inductively coupled laser ablation mass spectrometry. Applied Physics A.

[22] Basso E (2009). Composition of the base glass used to realize the stained glass windows by Duccio di Buoninsegna (Siena Cathedral, 1288–1289 AD): A geochemical approach. Materials Characterization.

[23] Garcia Vallès M, Vendrell Saz M (2002). The glasses of the transept’s rosette of the Cathedral of Tarragona: Characterization, classification and decay. Boletin de la Sociedad Española de Ceramica y Vidrio.

[24] Garcia Vallès M, Gimeno-Torrente D, Martínez-Manent S, Fernández-Turiel JL (2003). Medieval stained glass in a Mediterranean climate: Typology, weathering and glass decay, and associated biomineralization processes and products. American Mineralogist.

[25] Gulbiten O, Mauro JC, Guo X, Boratav ON (2018). Viscous flow of medieval cathedral glass. Journal of the American Ceramic Society.

[26] Zanotto ED (1998). Do cathedral glasses flow?. American Journal of Physics.

[27] Hunt RWG (1991). Measuring colour.

[28] Gratuze, B., Soulier, I., Barrandon, J.-N. & Foy, D. The origin of cobalt blue pigments in French glass from the thirteenth to the eighteenth centuries. In Hook, D. R., Gaimster, D. R. M. & British Museum (eds) *Trade and Discovery: The Scientific Study of Artefacts from Post-Medieval Europe and Beyond*, No. 109 in Occasional paper/British Museum, 123–133 (Department of Scientific Research, British Museum, London, 1995).

[29] Bontemps, G. *Guide du verrier: traité historique et pratique de la fabrication des verres*, *cristaux*, *vitraux*, English translation is edited by the Society of Glass Technology (Librairie du “Dictionnaire des arts et manufactures”, Paris, 1868).

[30] Bettembourg, J. Etude de verres bleus de vitraux. Analyse par spectrométrie d’absorption atomique. *Proceedings of the IX International Congress on Glass* 225–239 (1971).

[31] Villain O, Galoisy L, Calas G (2010). Spectroscopic and structural properties of Cr3+ in silicate glasses: Cr3+ does not probe the average glass structure. Journal of Non-Crystalline Solids.

[32] Calligaro T (2008). PIXE in the study of archaeological and historical glass. X-Ray Spectrometry.

[33] Cable M, Xiang Z (1989). Extinction coefficient of the cupric ion in soda-lime-silica glasses. Glastechnische Berichte.

[34] Weyl, W. *Coloured Glasses* (Society of Glass Technology, 1976).

[35] Vercamer V (2015). Diluted Fe3+ in silicate glasses: Structural effects of Fe-redox state and matrix composition. An optical absorption and X-band/Q-band EPR study. Journal of Non-Crystalline Solids.

[36] Bingham, K. & Parke, S. Absorption and Fluorescence Spectra of Divalent Manganese in Glasses. *Physics and Chemistry of Glasses* **6**, 224–& (1965).

[37] Terczynska-Madej A, Cholewa-Kowalska K, Laczka M (2010). The effect of silicate network modifiers on colour and electron spectra of transition metal ions. Optical Materials.

[38] Jackson CM (2005). Making Colourless Glass in the Roman Period. Archaeometry.

[39] Gliozzo E (2017). The composition of colourless glass: A review. Archaeological and Anthropological Sciences.

[40] Bingham P, Jackson C (2008). Roman blue-green bottle glass: Chemical–optical analysis and high temperature viscosity modelling. Journal of Archaeological Science.

[41] Möncke D, Papageorgiou M, Winterstein-Beckmann A, Zacharias N (2014). Roman glasses coloured by dissolved transition metal ions: Redox-reactions, optical spectroscopy and ligand field theory. Journal of Archaeological Science.

[42] Lagabrielle, S. & Velde, B. Evolution of stained glasses compositions during the middle ages - analyses and observations made on Cluny collection. *Annales du 16ème congrès AIHV* (2003).

[43] Chalmin E, Farges F, Brown GE (2009). A pre-edge analysis of Mn K-edge XANES spectra to help determine the speciation of manganese in minerals and glasses. Contributions to Mineralogy and Petrology.

[44] Long BT, Peters LJ, Schreiber HD (1998). Solarization of soda–lime–silicate glass containing manganese. Journal of non-crystalline solids.

[45] Pérez-Villar S, Rubio J, Oteo JL (2008). Study of color and structural changes in silver painted medieval glasses. Journal of Non-Crystalline Solids.

[46] Lautier, C. & Sandron, D. *Antoine de Pise: l*’*art du vitrail vers 1400* (CTHS, Comité des travaux historiques et scientifiques, 2008).

[47] Paynter, S. & Jackson, C. M. Mellow yellow: An experiment in amber. *Journal of Archaeological Science: Reports*, 10.1016/j.jasrep.2017.11.038 (2017).

[48] Kunicki-Goldfinger JJ (2014). Technology, production and chronology of red window glass in the medieval period – rediscovery of a lost technology. Journal of Archaeological Science.

[49] Pastoureau M (2000). Bleu histoire d’une couleur.

[50] Pastoureau M (2013). Vert. Histoire d’une couleur.

[51] Perrot F (1996). La couleur et le vitrail. Cahiers de Civilisation Médiévale.

[52] Gage, J. *Colour and Culture: Practice and Meaning from Antiquity to Abstraction* (Thames and Hudson, 1993).

[53] Trichereau B, Loisel C (2017). The nineteenth-century restoration processes of Louis Germain Vincent-Larcher at the Cathedral of Troyes. Glass Technology-European Journal of Glass Science and Technology Part A.

[54] Balcon-Berry, S., Pastan, E. & Minois, D. Vincent-Larcher et les vitraux de la cathédrale de Troyes: un face-à-face de quarante ans. In *Regards sur le vitrail du XIXe siècle*. *L*’*œuvre de Louis Germain Vincent-Larcher*, 143–164 (Conseil départemental de l’Aube/Snoeck, 2017).

