# Supplementary material for: The Grande Rose of the Reims Cathedral: an eight-century perspective on the colour management of medieval stained glass
Source: Sci Rep. 2019 Mar 1;9:3287. doi: 10.1038/s41598-019-39740-y (PMC6397263; doi:10.1038/s41598-019-39740-y)
Supplement: Supplementary file 1 — Supplementary Information [file 41598_2019_39740_MOESM1_ESM.pdf]

Supplementary materials for The *Grande Rose* of Reims cathedral: an eight-century perspective on the colour management of medieval stained glass

Natan Capobianco[1,*], Myrtille O.J.Y. Hunault[2], Sylvie Balcon-Berry[3], Laurence Galoisy[1], Dany Sandron[3], and Georges Calas[1]

[1]Sorbonne Université, Muséum National d'Histoire Naturelle, UMR CNRS 7590, IRD, Institut de Minéralogie, de Physique des Matériaux et de Cosmochimie, IMPMC, 75005 Paris, France
[2]SOLEIL synchrotron, L'Orme des Merisiers Saint-Aubin BP48, 91192 Gif-sur-Yvette, France
[3]Sorbonne Université, UMR CNRS 8150, Centre André Chastel, 75005 Paris, France
[*]natan.capobianco@sorbonne-universite.fr
[+]these authors contributed equally to this work

1 The Grande Rose of Reims

1.1 Historical background

The Cathedral of Reims (France), registered in 1991 as a UNESCO World Heritage Site, is a masterpiece of the medieval Gothic architecture, which spread throughout Europe from Northern France from the end of the 11th century until Renaissance. The site has been an important place of Christian worship since the 5th century and hosted the baptism of king Clovis in 496, which made him the first Christian King of the Franks. Since the 9th century, the cathedral has been the place of coronation of almost every King of France, which made the cathedral and the city of Reims an important historical and symbolic place of the kingdom, as shown by the coronation of Charles VII after Jeanne d'Arc conquered the city in 1429 during the Hundred Years' War[1].

The building of the current cathedral of Reims took place from 1211 after the burning of the previous Carolingian cathedral and has been completed during the 14th century, while following the original design that gives the cathedral a unity of style, which is unusual in Northern France. The building is thus posterior to other great French cathedrals like Paris (beginning in 1163) and Chartres (in 1194) but precedes the ones of Amiens (in 1220) and Beauvais (in 1230). The Grande Rose from the western façade (12.5 m of diameter) was built around 1275. Storms and damages to the cathedral made restoration necessary as soon as 1481. In 1911, restoration work by the Simon-Marcq workshop (based in Reims) resulted in the replacement of almost every non-medieval glass piece. Another campaign took place in 1920 to repair World War I damages, especially on the northern part, which was damaged by an important fire on September 19th 1914[1]. The Rose has not been modified since then in spite of numerous works occurring on the Cathedral in the latest decades. In 2015, every panel from the rose has been taken out for restoration, allowing us to perform for the first time the physico-chemical study of these stained glass windows.

1.2 Restoration

The restoration was carried out by the France Vitrail workshop in Neuville-sur-Sarthe, near Le Mans, in France. The panels went through consolidation of the lead network and soft cleaning of the glasses (removal of the dirt layer). After the restoration, we analysed 101 pieces of glass from 20 different panels (the studied panels are given on Figure S1, repartition of analysed glasses by colour and age is summarised on Figure S2, detailed count given on Table S1). The analysed glass pieces were chosen based on their colour and estimated date of fabrication in order to collect the most exhaustive corpus. The latest restoration work from 1911 ensures that most of glass pieces are either modern from 1911 or 1920 or original from the 13th century[1]. This was confirmed by the critical observation which determined the age of the glasses upon visual analysis of the glass and the paintings and comparison with other well-known glasses. Two colourless samples peculiarly well preserved have been identified as from the 12th century. The Northern half of the Rose has been severely damaged by the fire of the Northern tower in September 1914 and contains modern glasses in majority. Therefore, most of the samples that were analysed come from the Southern half of the Rose, which contains a higher number of original glasses than the other half.

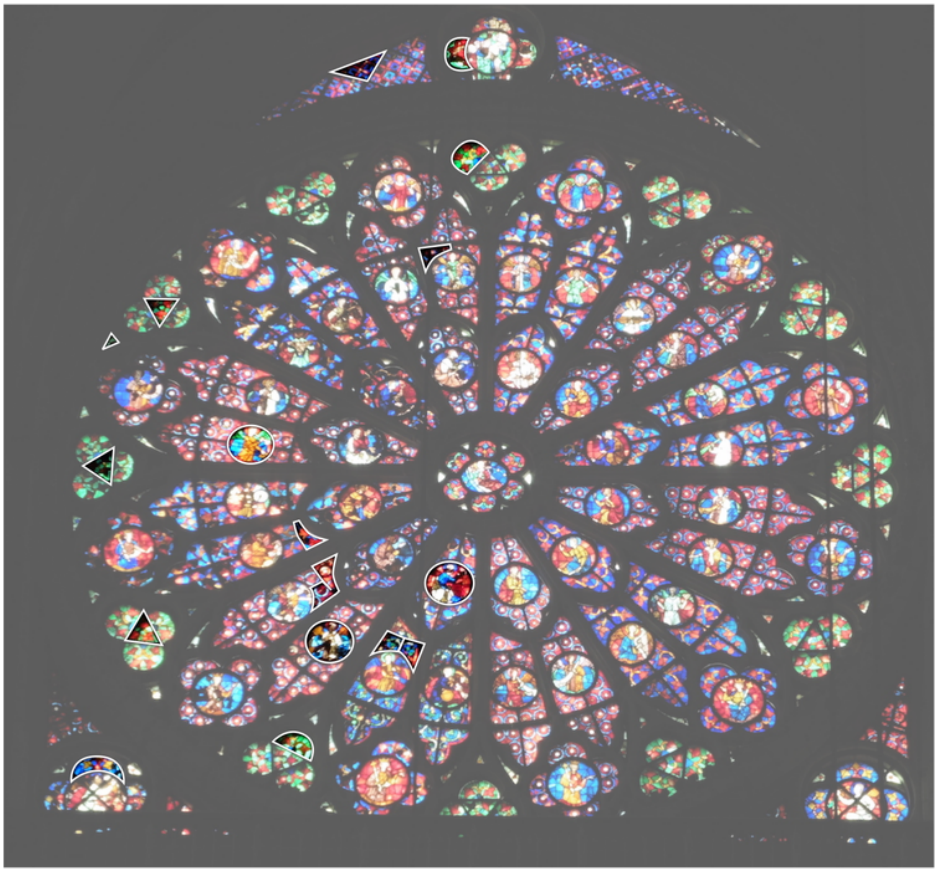

Supplementary Figure S1. Panels circled in white were studied. Further two panels are not seen in this image.

	Blue	Colourless	Yellow	Purple	Red	Green	Total
12th century	0	2	0	0	0	0	2
13th century	11(+8)	5(+6)	1(+4)	2(+2)	3	14(+9)	36(+29)
20th century	15(+17)	6(+1)	4(+2)	5	2	16(+9)	48(+29)
Unknown	2	0	5	0	2	6	15
Total by colour	28(+25)	13(+7)	10(+6)	7(+2)	7	36(+18)	101(+58)

Supplementary Table S1. Number of glasses analyzed in colorimetry (+ in thickness) by colour and by age.

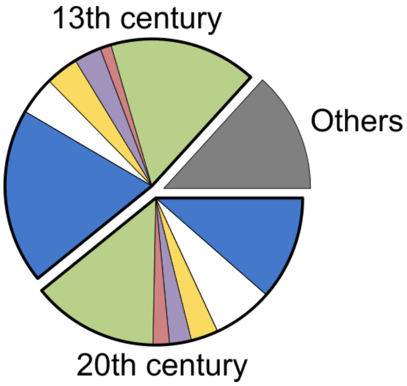

Supplementary Figure S2. Colour repartition of the analysed glasses according to dating. Detailed count is given Table S1

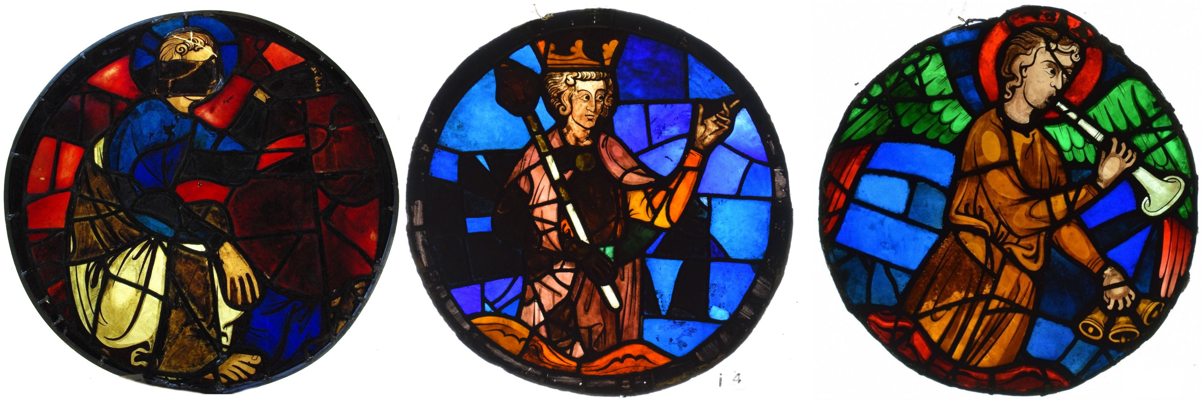

(a) H1 panel **(b)** I4 panel **(c)** K2 panel

Supplementary Figure S3. Example of three panels which show the variety of blue colours that are placed next to each other

1.3 The style of the Rose

As sketched in Figure 1d (main text), the Rose consists in 12 "petals" that assemble around a window representing Mary. Each petal has 4 circular figurative panels, representing one character each, arranged in 3 rings: 12 apostles (inner ring), 24 musician angels (middle ring) and 12 kings (outer ring). There are two kind of petals, which strictly alternate: petals whose characters have blue background, have their other panels with red oculi and petals whose characters have red background, have their other panels with plant-covered ornaments on blue backgrounds. On the outermost ring, trefoils with plants on green background are inserted between the petals. Such use of green glass was seldom in France in Middle-Age[2,3]. This peculiar use of green colour on the outermost ring may be explained by the stone ring of sculptures of plants around the rose, which were probably originally painted. The green colour of the rose at the border may have formed a transition between the outer stones and the depicted program of the stained glasses. Plants usually have red leaves and yellow stems.

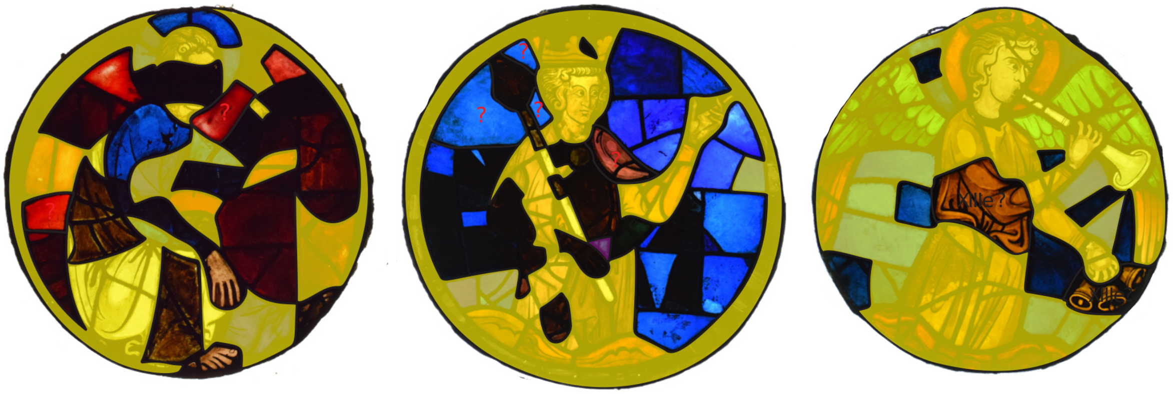

(a) H1 panel **(b)** I4 panel **(c)** K2 panel

Supplementary Figure S4. Authenticity screening of the panels from previous figure: glasses shaded in yellow are identified as modern, glasses with no shade are identified as dating from 13th century.

2 Measuring the spectrum and the colour

2.1 Experimental device

See scheme in Figure S5

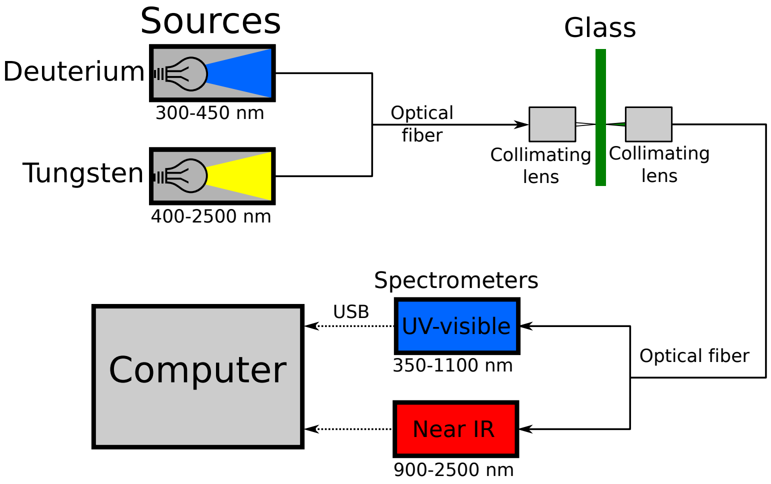

Supplementary Figure S5. Experimental set-up of the portable spectrometer.

2.2 Importance of the deuterium lamp

As shown in Figure S6, without the deuterium lamp, the values of the absorption are underestimated under 400 nm. The adding of the deuterium lamp allows to measure the spectrum with a good quality up to 370 nm. The better measurement of the blue/purple part (380-450nm) of the spectrum allows better calculation of colorimetric coordinates.

For sample which are highly absorbent 9000-11000 cm^{-1} range, part of the spectra in this range tends to be noisier than the rest of the spectrum because of the low sensitivity of the spectrometers in this range of energies.

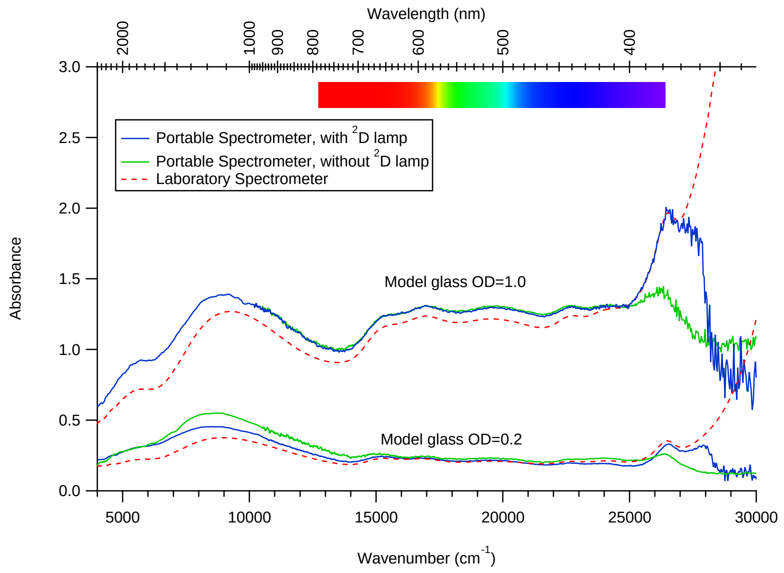

Supplementary Figure S6. Spectra of model glasses of optical density 0.2 and 1.0, acquired with the portable spectrometer with and without the deuterium lamp and with a standard laboratory spectrometer (Perkin Elmer)

2.3 Choice of the colorimetric system

We chose to calculate the colorimetric coordinates in the Yxy colorimetric system, rather than in the $L^*a^*b^*$ colorimetric system. Two reasons lead to this choice: First, the Yxy system is less sensitive to the absorption background (due to the surface alteration of the glass) than the $L^*a^*b^*$ system: the x and y coordinates are independent from a constant background. Second, the Yxy system allows plotting the colour on a two-dimensional plot, whereas the interpretation of L*a*b* coordinates in terms of colour requires considering the three dimensions [Hunt]. In a xy plot, the lost third dimension (Y) is the total luminance, which does not alter the hue nor the saturation of the resulting colour, but its value (thus the brightness).

In the Yxy colorimetric system, three functions $\bar{x}$, $\bar{y}$ and $\bar{z}$ are defined. These three function are the colorimetric functions of the standard observer CIE 1931. The shapes of the functions are given in Figure S7. The colorimetric coordinates X, Y and Z are then computed by projecting on each colorimetric function the product of the optical spectrum and the standard illuminant (here the D65 illuminant, average midday light in Western Europe).

$$\begin{cases} X = K \int_\lambda S(\lambda) I(\lambda) \overline{x}(\lambda) d\lambda \\ Y = K \int_\lambda S(\lambda) I(\lambda) \overline{y}(\lambda) d\lambda \\ Z = K \int_\lambda S(\lambda) I(\lambda) \overline{z}(\lambda) d\lambda \end{cases}$$

Where $S(\lambda)$ is the optical spectrum and $I(\lambda)$ is the power distribution of the illuminant. K is a normalisation factor, so that Y is the total brightness.

x, y and z are then obtained by normalising X, Y, Z so that $x+y+z=1$.

$$\begin{cases} x = \frac{X}{X+Y+Z} \\ y = \frac{Y}{X+Y+Z} \\ z = \frac{Z}{X+Y+Z} \end{cases}$$

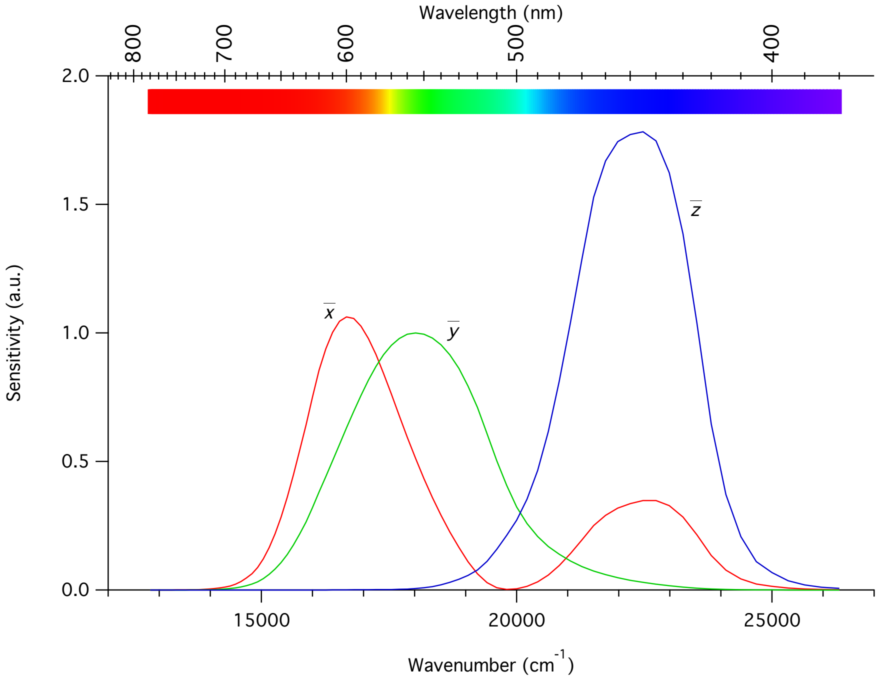

Supplementary Figure S7. Colorimetric functions of the standard observer CIE 1931

2.4 Influence of glass thickness on colour

	Blue	Colourless	Yellow	Purple	Red	Green
20th century	3.16	2.93	2.60	2.81	2.69	2.85
13th century	2.88	3.13	2.73	3.20	3.58	3.07

Supplementary Table S2. Average thicknesses (in mm)

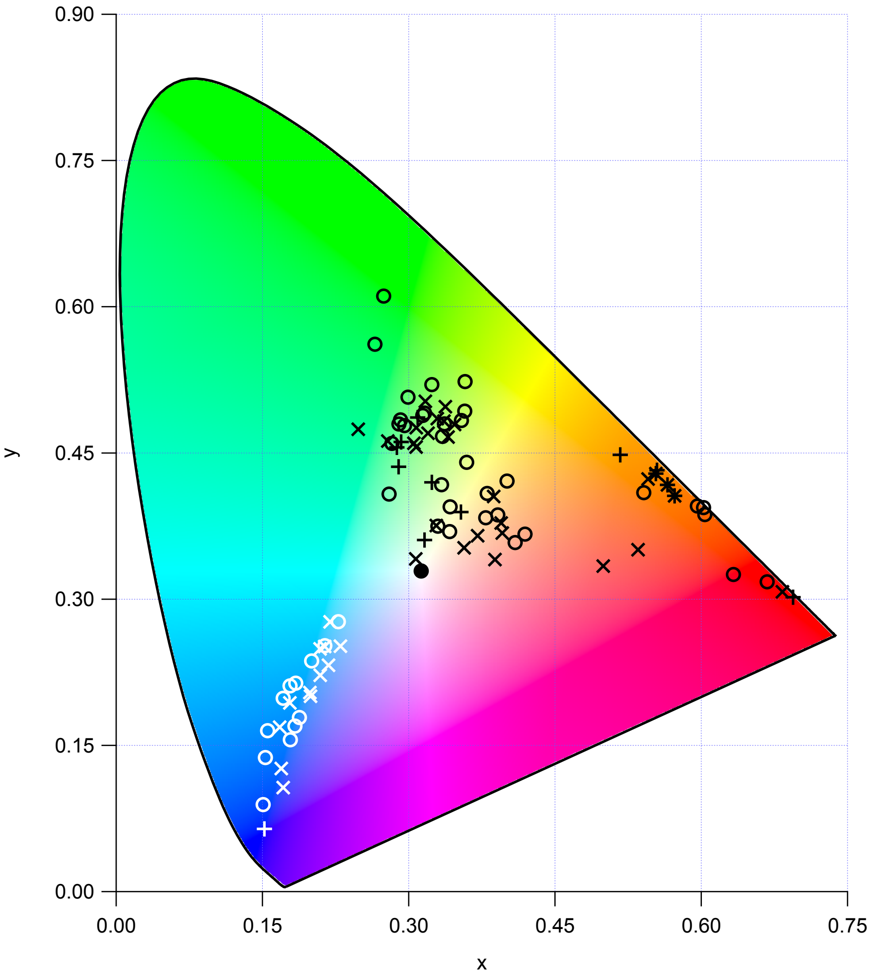

Supplementary Figure S8. CIExy chromaticity diagram of the colour of all the samples, recalculated after normalizing their thickness to 3.0 mm. ○: modern glasses, ×: medieval glasses. Blue glasses have a white marker for legibility reasons. The black point in the centre have the coordinates of the white point of the diagram.

2.5 Estimation of the concentration

For the samples with only one colouring species, absorption is read off at the maximum and a lower value of absorption of the spectrum normalized by thickness.

Concentration is then estimated thanks to the Beer-Lambert law:

$$A(\lambda) = l * c * \varepsilon(\lambda) \qquad \text{thus} \qquad c = \frac{A_{max} - A_{min}}{l(\varepsilon_{max} - \varepsilon_{min})}$$

As we measured the absorbance on a normalized spectrum, $l = 1$cm and thus l will be omitted in the following equations. The values of absorption coefficient at λ_{max} and λ_{min} are given in Table S3.

When two colourants a and b are present, absorption is read off at wavelength. Concentration of both species is then calculated by solving the following system of equation:

$$\begin{cases} A_1 - A_3 = c^a(\varepsilon_1^a - \varepsilon_3^a) + c^b(\varepsilon_1^b - \varepsilon_3^b) \\ A_2 - A_3 = c^a(\varepsilon_2^a - \varepsilon_3^a) + c^b(\varepsilon_2^b - \varepsilon_3^b) \end{cases}$$

Number in subscript is relative to the wavelength, letter in superscript is relative to the colouring ion. Values of absorption coefficient used for the calculations are given in Table S4

[1] In the case of cobalt, the first value refers to the absorption coefficient in soda-lime silica glasses, the second to potash-lime silica glasses. We assumed that modern glasses had soda-lime composition whereas medieval glasses had potash-lime.

	λ_{max}	$\varepsilon(\lambda_{max})$	λ_{min}	$\varepsilon(\lambda_{min})$	Source
Fe^{2+}	900	8.4	550	0.6	4
Co^{2+}	590	33.6/46.4[1]	800	1.1/1.5[1]	5
Cu^{2+}	800	9.0	540	1.8	6
Mn^{3+}	490	7.9	1000	0.2	7
Cr^{3+}	450	6.4	550	1.0	4

Supplementary Table S3. Wavelengths (in nm) and corresponding absorption coefficients (in $cm^{-1}\,w\%^{-1}$) used for the estimation of concentration of ions in glass coloured by only one colourant. For conversion of ε, glass density has been approximated at 2.4

a	b	λ_1	ε_1^a	ε_1^b	λ_2	ε_2^a	ε_2^b	λ_3	ε_3^a	ε_3^b
Cr^{3+}	Cu^{2+}	450	6.4	0.6	800	0.5	9.0	540	1.2	1.8
Co^{2+}	Cu^{2+}	590	33.6/46.4[1]	3.9	800	1.1/1.5[1]	9.0	460	3.8/5.2[1]	0.6
Co^{2+}	Fe^{2+}	590	33.6/46.4[1]	1.0	900	1.4/1.9[1]	8.4	460	3.8/5.2[1]	0.0

Supplementary Table S4. Wavelength (in nm) and corresponding absorption coefficient (in $cm^{-1}\,w\%^{-1}$) used for the estimation of concentration of ions in glass coloured by each pair of colourants.

2.6 Dependency of highest energy Co^{2+} peak with Cu^{2+} content

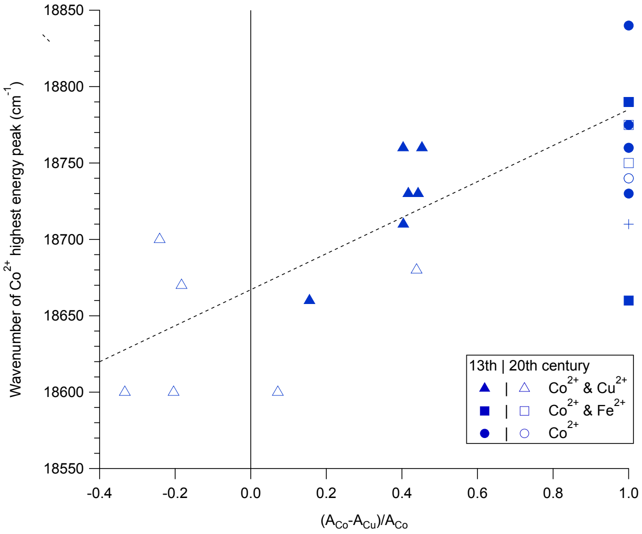

Supplementary Figure S9. Plot that illustrate the variation of the position of Co^{2+} highest energy peak which the influence of Cu^{2+} in the OAS.

Figure S9 shows the variation of the position of Co^{2+} highest energy peak (around 535 nm) with the influence of Cu^{2+} in the OAS of blue glasses. The bottom axis is by applying the following formula:

$$B = \frac{\text{Absorbance at } Co^{2+} \text{ maximum (590 nm)} - \text{Absorbance at } Cu^{2+} \text{ maximum (800 nm)}}{\text{Absorbance at } Co^{2+} \text{ maximum (590 nm)}}$$

For a sample without Cu^{2+}, B is taken equal to 1, as absorption of Co^{2+} is negligible at 800 nm and absorption of iron is negligible at 590 nm. The higher the Cu2+ content, the lower will be B. Thus B quantifies the relative influence of Cu2+ absorption in the spectrum.

3 Details on plots from the main contents

3.1 Zoom on Figure 6a

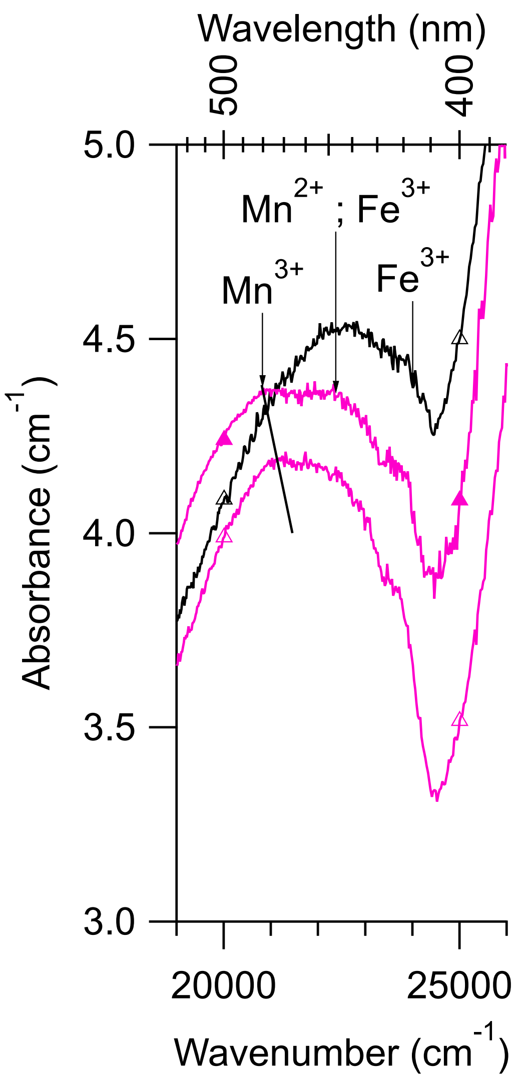

Supplementary Figure S10. Zoom on the 19000-26000 cm^{-1} range of Fig. 6a.

3.2 Smoothing of Fe^{2+} band in Figure 3 (main text)

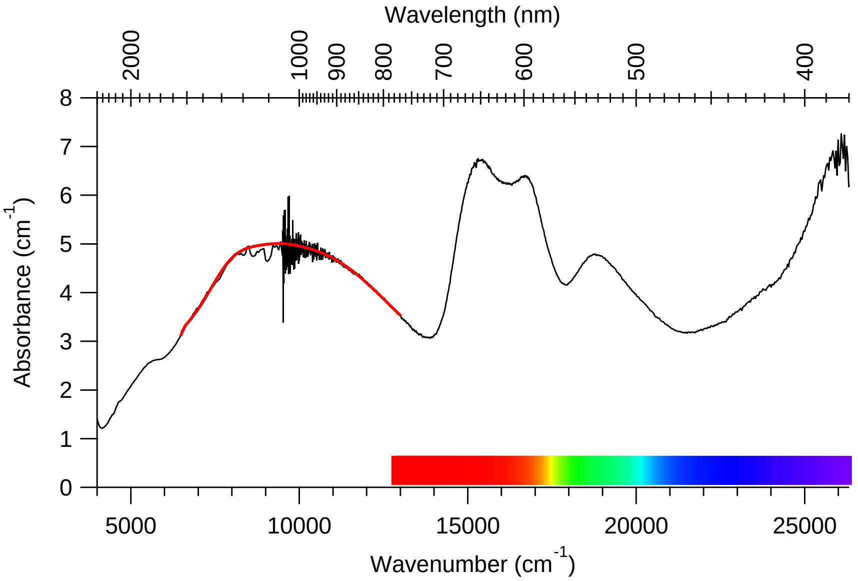

Supplementary Figure S11. Comparison between the original and the smoothed spectrum of the medieval Co^{2+}-Fe^{2+} sample in Figure 4 (main text)

References

1. Demouy, P. & Jordan, T. *Reims - La grâce d'une cathédrale* (La Nuée bleue, Strasbourg, 2010).
2. Grodecki, L. & Brisac, C. *Le Vitrail Gothique Au 13e Siècle* (Office du Livre, Paris, 1984).
3. Lafond, J. & Perrot, F. *Le Vitrail: origines, technique, destinées* (La Manufacture, 1988).
4. Bamford, C. R. *Colour Generation and Control in Glass* (Elsevier Amsterdam, 1977).
5. Hunault, M. *Rôle Des Éléments de Transition (Co, Cu) Dans La Coloration Des Verres. Application Aux Vitraux Du Moyen-Âge.* Ph.D. thesis, UNIVERSITE PIERRE ET MARIE CURIE–PARIS (2014).
6. Singh, S. P. & Kumar, A. Molar extinction coefficients of the cupric ion in silicate glasses. *J. Mater. Sci.* **30**, 2999–3004 (1995). DOI 10.1007/BF00349674.
7. Möncke, D., Papageorgiou, M., Winterstein-Beckmann, A. & Zacharias, N. Roman glasses coloured by dissolved transition metal ions: Redox-reactions, optical spectroscopy and ligand field theory. *J. Archaeol. Sci.* **46**, 23–36 (2014). DOI 10.1016/j.jas.2014.03.007.
